# The Impact of Impurity Gases on the Hydrogen Embrittlement Behavior of Pipeline Steel in High-Pressure H_2_ Environments

**DOI:** 10.3390/ma17092157

**Published:** 2024-05-05

**Authors:** Chengshuang Zhou, Hongbin Zhou, Lin Zhang

**Affiliations:** College of Materials Science and Engineering, Zhejiang University of Technology, Hangzhou 310014, China; zhoucs@zjut.edu.cn (C.Z.); 13657033637@163.com (H.Z.)

**Keywords:** L360 steel, mixed gases, fatigue crack growth, hydrogen permeation

## Abstract

The use of hydrogen-blended natural gas presents an efficacious pathway toward the rapid, large-scale implementation of hydrogen energy, with pipeline transportation being the principal method of conveyance. However, pipeline materials are susceptible to hydrogen embrittlement in high-pressure hydrogen environments. Natural gas contains various impurity gases that can either exacerbate or mitigate sensitivity to hydrogen embrittlement. In this study, we analyzed the mechanisms through which multiple impurity gases could affect the hydrogen embrittlement behavior of pipeline steel. We examined the effects of O_2_ and CO_2_ on the hydrogen embrittlement behavior of L360 pipeline steel through a series of fatigue crack growth tests conducted in various environments. We analyzed the fracture surfaces and assessed the fracture mechanisms involved. We discovered that CO_2_ promoted the hydrogen embrittlement of the material, whereas O_2_ inhibited it. O_2_ mitigated the enhancing effect of CO_2_ when both gases were mixed with hydrogen. As the fatigue crack growth rate increased, the influence of impurity gases on the hydrogen embrittlement of the material diminished.

## 1. Introduction

Hydrogen is an excellent energy carrier. It is characterized by a high energy content and the absence of pollution [1]. Among the various hydrogen transportation methods, pipelines are advantageous because of their large capacity and low cost [2,3,4]. Considering the high costs associated with constructing dedicated hydrogen pipelines, using existing natural gas pipelines for the transport of hydrogen or hydrogen-blended natural gas is a viable option. Generally, materials in service are prone to stress concentrations at certain locations, particularly under cyclic loading stresses. This leads to crack initiation. These cracks are precursors to metal workpiece failures. The presence of different environmental media significantly influences crack initiation and propagation. The mechanical properties of metal materials deteriorate in hydrogen-containing conditions because of the inevitable ingress of hydrogen atoms. This results in premature failure and, consequently, hydrogen embrittlement [5,6,7,8,9].

The hydrogen embrittlement process in metal materials generally involves the following steps [10,11,12,13]: (1) hydrogen molecules are physically adsorbed on the material surface and chemically dissociate to produce hydrogen atoms; (2) atomic hydrogen diffuses into the interior of the material; (3) the diffused hydrogen atoms accumulate at defects or microcracks within the crystal structure of the specimen itself when the specimen is subjected to stress, reducing the bond strength between metal atoms because of the interaction between hydrogen and the metal atoms; (4) this then leads to the premature failure of the material. This idealized scenario is closely related to the expansion of hydrogen-assisted cracks in a high-purity hydrogen environment. There is convincing evidence that the presence of certain gaseous species in hydrogen—namely, molecules composed of carbon, oxygen, and sulfur with unsaturated chemical bonds—can inhibit the embrittlement process [14,15]. The inhibitory effect of these gaseous species is attributed to the following two characteristics: (1) the molecules adsorb on the steel surface in a dissociated chemical adsorption manner, and (2) carbon, oxygen, and sulfur atoms on the steel surface hinder the absorption of hydrogen. Inhibitors disrupt the sequence of interactions between hydrogen and the material, promoting hydrogen embrittlement before it takes place. Thus, hydrogen-assisted crack propagation is inhibited in gas environments containing these inhibitors. Natural gas contains many impurity gases, including O_2_, N_2_, CO, CO_2_, H_2_S, and SO_2_. These gases can act as inhibitors or catalysts during the adsorption process of hydrogen. Understanding the impact mechanisms of impurity gases on the hydrogen embrittlement behavior of hydrogenated natural gas pipelines is, therefore, of significance.

Research by Nelson et al. [15] conclusively demonstrated that oxygen effectively inhibited the hydrogen-induced acceleration of fatigue crack propagation in steel. Similarly, Michler and colleagues [16] observed that low-pressure oxygen at an ambient temperature suppressed the initiation and propagation of hydrogen-accelerated fatigue cracks in steel, but this inhibitory effect significantly weakened as the temperature decreased and hydrogen pressure increased. Somerday and others [17] quantified the impact of mechanical and environmental variables on the inhibitor-modified acceleration of fatigue crack propagation in steel in hydrogen environments and provided experimental results on the fatigue crack propagation of X52 pipeline steel in oxygen-containing H_2_ gas. They elucidated the mechanisms behind the effectiveness or ineffectiveness of oxygen from the perspective of the competitive re-passivation rates of a fresh iron surface as well as the rates of fatigue crack propagation. At relatively lower fatigue crack propagation rates, the addition of O_2_ to H_2_ gas inhibited hydrogen-accelerated fatigue crack propagation. The inhibitory effect of O_2_ vanished when the fatigue crack propagation rate exceeded a critical value. At lower fatigue crack propagation rates, oxygen had sufficient time to cover the newly formed surface because of the slower rate of new-surface formation, thereby impeding hydrogen absorption.

Studies have demonstrated that CO_2_ promotes the fatigue crack propagation of pure iron in H_2_ environments [18,19]. Hydrogen permeation tests were conducted in these studies. The results revealed that CO_2_ reduced the time for hydrogen to penetrate but without a positive effect on the steady-state current. This indicated that CO_2_’s promotion of hydrogen embrittlement was related to a reduction in its surface interaction time. CO_2_ has been observed to lower the energy barrier for hydrogen atom diffusion from the surface to the sub-surface of iron, which is key to CO_2_’s promotion of hydrogen embrittlement in hydrogen environments. CO_2_, especially at lower ΔK values, increases the concentration of hydrogen at the crack tip, reduces dislocation migration around the crack, and diminishes plastic deformation around the crack. This increases the crack propagation rate and promotes hydrogen embrittlement behavior.

Previous experiments have mainly focused on the impact of single gas species on the hydrogen embrittlement behavior of pipeline materials in high-pressure hydrogen environments. Fewer studies have explored the effects of mixed gases. The introduction of certain proportions of mixed gases could potentially inhibit the hydrogen embrittlement of materials whilst reducing purification costs. Exploring the mechanisms by which multiple impurity gases affect the hydrogen embrittlement behavior of pipeline steel in high-pressure H_2_ environments is of significance to enhance the safety and economic efficiency of hydrogen-blended natural gas pipeline networks.

In this study, we investigated the impact of impurity gases on the fatigue crack propagation of L360 steel in a hydrogen environment under high-pressure gas conditions. We explored the influence of gas adsorption on the material surface and hydrogen permeation through hydrogen permeation experiments. We examined the fatigue crack morphology and material fracture morphology using scanning electron microscopy and analyzed the effect of impurity gases on the rate of hydrogen-accelerated crack propagation.

## 2. Materials and Methods

### 2.1. Experimental Materials

**Figure 1:**
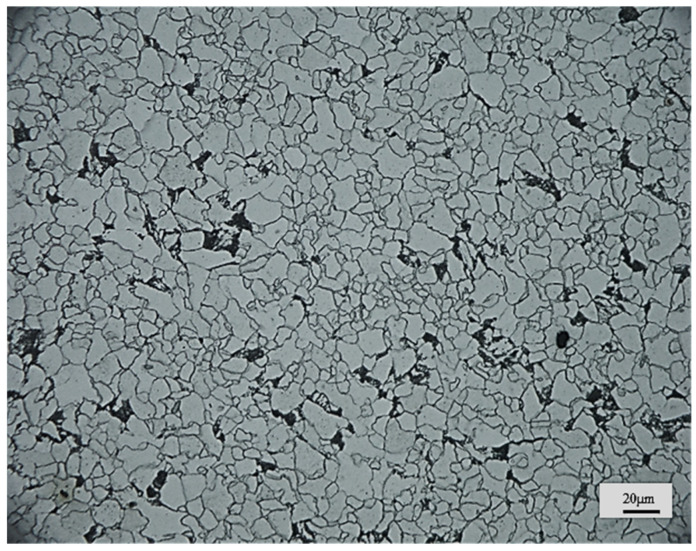
Microstructure diagram of L360 steel. Magnification: 500×.

The selected steel material for this study was designated as X52, which is also known as L360 and. The material’s yield strength was identified as 370 MPa, and its tensile strength was 450 MPa. Table 1 displays the chemical composition of L360 steel. Figure 1 presents a metallographic image of L360; the material primarily consisted of a ferritic structure with a sprinkling of pearlite at the grain boundaries characteristic of a low-carbon steel composition.

**Figure 2:**
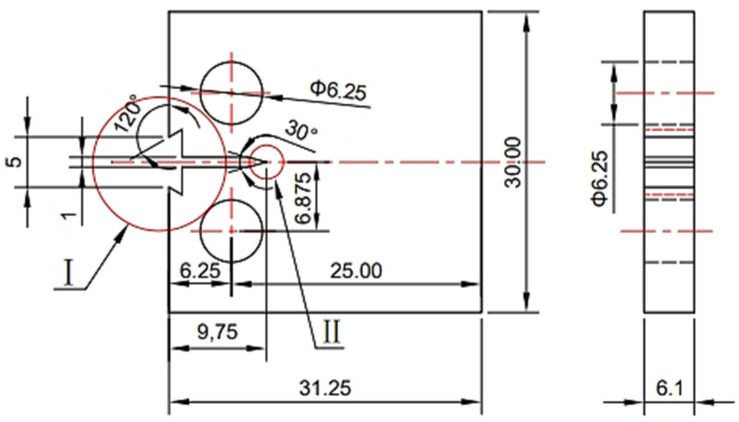
Compact tensile specimens for fatigue crack propagation experiments (CT specimen).

The original steel material was cut into smaller pieces parallel to the rolling direction to facilitate the subsequent processing. According to the ASTM-E647 standard [20], these materials were then fashioned into compact tension specimens (CT specimens) with a thickness of 6 mm for the fatigue crack growth experiments following the dimensions provided in Figure 2. The prepared CT specimens underwent a sequence of polishing treatments. We initially employed silicon carbide sandpapers of 240, 600, 1000, 1500, and 2000 grit (in sequence) for sample grinding. This was followed by progressive polishing using diamond pastes of 2.5 microns, 1.0 micron, and 0.5 microns.

### 2.2. Fatigue Crack Propagation Experiment

**Figure 3:**
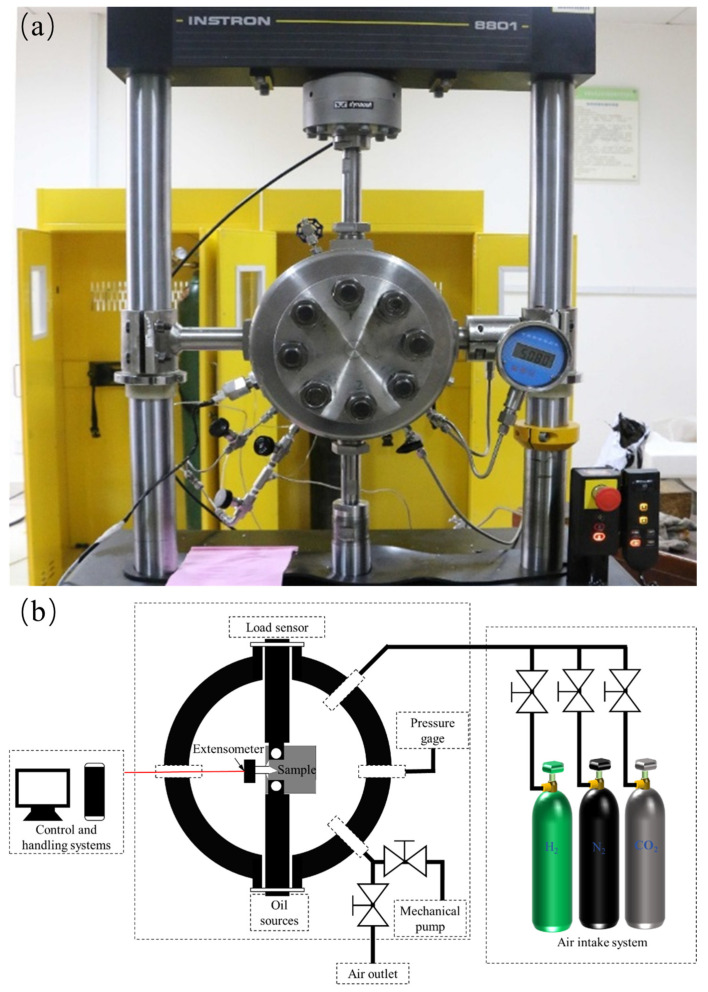
Schematic diagram of fatigue test equipment. (**a**) Instron fatigue testing machine; (**b**) experimental system schematic diagram.

The fatigue crack propagation test was conducted in accordance with the ASTM-E647 standard [20]. The specific dimensions of the samples are depicted in Figure 2. The experimental apparatus used was an Instron 8801 (100 KN) universal testing machine (Armstrong (Shanghai) Testing Equipment Trading Co., Ltd.—China Headquarters, Shanghai, China) with a constant load amplitude testing (ΔP constant) method. The testing conditions were as follows: ΔP = 5 KN; a stress ratio (R) of 0.1; a Poisson’s ratio (ν) of 0.3; and a modulus of elasticity (E) of 2.06 E5 MPa. All of the specimens were uniformly pre-cracked in air to a length of 6 mm with a final crack length of 12 mm. The gases taken in the experiment are all high-purity gases with a level of 5N, and the gas mixture is mixed in the environment box according to the way of passing O_2_ first, adding CO_2_, and finally adding H_2_, and the content of the gases is observed by the indication of the pressure gauge. Prior to the commencement of the experiment, the samples were permitted to remain in an environment comprising a mixture of gases for a period of two hours. In accordance with the pertinent standards and the actual circumstances of engineering application, which take into account factors such as safety, demand and cost, a hydrogen pressure of 6 MPa was selected [4]. A 2.5% CO_2_ partial pressure significantly promotes hydrogen embrittlement in pure iron materials [18,21]. Additionally, a concentration of 350 ppm O_2_ has been identified as an inhibitor of this phenomenon, while a concentration of 35 ppm O_2_ has been identified as having a minimal effect on the embrittlement of materials under the aforementioned pressure system of 6 MPa. The compositions of the mixed gases are provided in Table 2. The experimental apparatus is presented in Figure 3.

### 2.3. Fatigue Crack and Fractography Analyses

The microscopic morphology of the transverse fracture path of the samples was examined using a scanning electron microscope (SEM, FEINOVA450, Thermo Fisher Scientific, Waltham, MA, USA). The previously inspected CT specimens were then pulled apart along the direction of the crack using an Instron 8801 (100 KN) universal testing machine to observe the fracture surface morphology formed during the prior fatigue crack propagation experiment. The operating voltage was set at 15 kV.

### 2.4. Hydrogen Permeation Experiment

**Figure 4:**
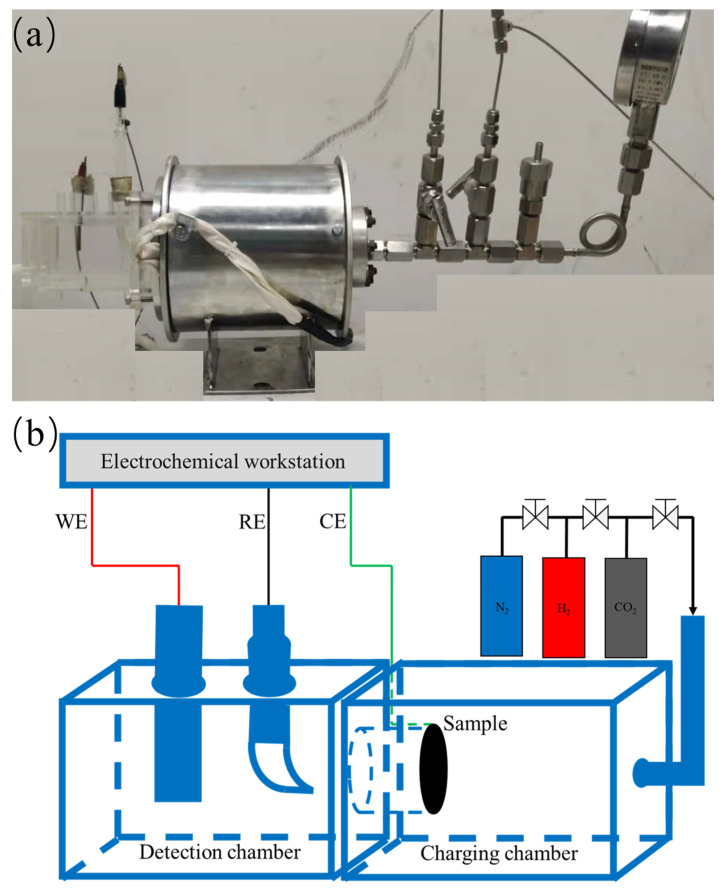
Schematic diagram of gas hydrogen permeation test equipment. (**a**) Hydrogen permeation equipment physical diagram; (**b**) Schematic diagram of hydrogen permeation equipment.

The hydrogen permeation experiment was primarily conducted to discern the interactions that transpired between hydrogen gas or a mixture of hydrogen and other gases with the metal surface. The test specimens for hydrogen permeation were coated with a nickel film on the detection side using a Watts electrolyte solution (comprising 250 g/L NiSO_4_, 45 g/L NiCl, and 40 g/L HBO_3_) at a plating temperature of 50 °C. The plating current density was 5 mA/cm^2^ with a plating duration of 5 min. The specimens were discs with a diameter of 3 cm and a thickness of 2 mm; these were sequentially polished with sandpapers of 240, 600, 1000, and 2000 grit until a scratch-free mirror finish was achieved. After single-sided nickel plating in a glove box, the surface was pickled before the specimen was mounted. The hydrogen charging side was exposed to pure hydrogen gas with a purity of 99.999% at 6 MPa. The detection side was placed in a 0.2 mol/L NaOH solution. The experiment was conducted at a temperature of 25 °C. Before testing, the hydrogenation vessel was purged with nitrogen and then evacuated to a vacuum with a pump. This purge–vacuum cycle was repeated 3–5 times to eliminate any residual gases adhering to the interior walls of the container. Background levels were reduced until the background current density fell below 0.1 μA/cm^2^ before commencing the experiment. The experimental setup is demonstrated in Figure 4.

## 3. Results

### 3.1. Fatigue Crack Growth Rate Curves

**Figure 5:**
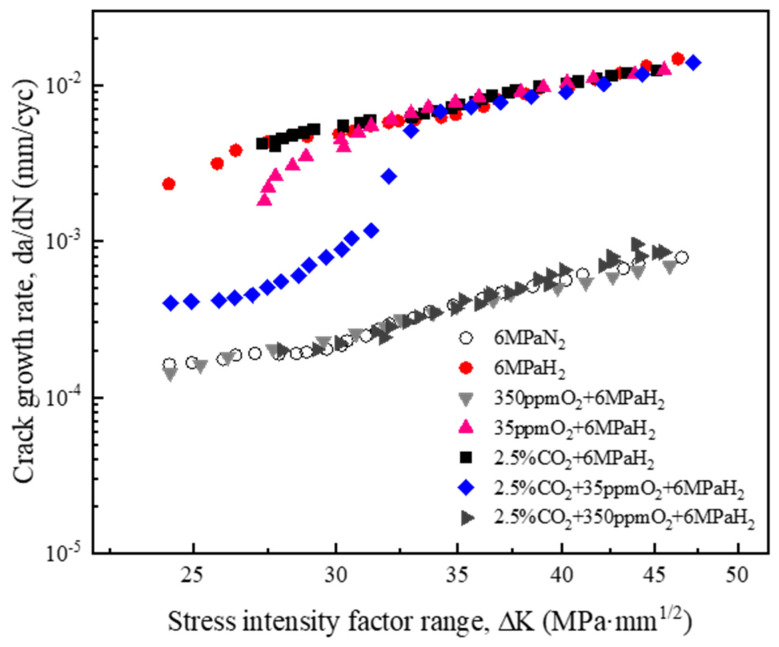
Fatigue crack growth (FCG) rate curves of L360 pipeline steel in various environments.

Figure 5 presents the fatigue crack propagation (FCG) rate curves for L360 pipeline steel in various environments (6 MPa N_2_, 6 MPa H_2_, 6 MPa H_2_ + 2.5% CO_2_, 6 MPa H_2_ + 350 ppm O_2_, and 6 MPa H_2_ + 35 ppm O_2_). ΔK represents the range of the stress intensity factor. We observed that the FCG rate in an H_2_ atmosphere was significantly elevated compared with that in an N_2_ atmosphere. This increase was potentially attributable to the interactions between hydrogen atoms and the crack tip, as suggested by prior analyses [22,23]. It has been substantiated [18] that CO_2_ exacerbates the hydrogen embrittlement of materials, with even a 2.5% concentration exerting a substantial effect. Our experiment incorporated 2.5% CO_2_ to demonstrate the facilitating role of CO_2_ on the hydrogen embrittlement of our material, and we observed FCG rates that were akin to those in a pure H_2_ environment. Comparatively, the fatigue crack growth curves within the 350 ppm O_2_ atmosphere approximated those in an N_2_ environment. This may have been due to the formation of an adequate oxygen layer over nascent crack surfaces, thereby impeding the interaction between hydrogen atoms and the crack tip [24,25]. Conversely, the incorporation of 35 ppm O_2_ yielded FCG rates that were closer to those observed under a pure hydrogen atmosphere. This may have been because the low oxygen content failed to thoroughly cover the newly formed surface, thereby not fully suppressing the acceleration of crack propagation by hydrogen atoms at the crack tip. At lower values of ΔK, the corresponding FCG curves were reduced. This suggested that at a low ΔK, oxygen played a role in restraining the acceleration of fatigue crack expansion caused by hydrogen [26,27].

Although the singular hydrogen embrittlement effects of CO_2_ and O_2_ are well documented, their synergistic interaction (i.e., if CO_2_ can veil the inhibitory effect of O_2_ or if O_2_ can dampen CO_2_’s facilitative action) remains an enigma. Figure 3 reveals our experimental findings. The FCG rates under 6 MPa H_2_, both with and without an admixture of 350 ppm O_2_, were comparable in the presence or absence of 2.5% CO_2_. This indicated that the presence of CO_2_ had a negligible impact on the hydrogen embrittlement of L360 pipeline steel under a high-pressure hydrogen environment with 350 ppm O_2_. The fatigue crack growth curves at lower stress intervals (20~30 MPa·m^1/2^) under 6 MPa H_2_ + 2.5% CO_2_ + 35 ppm O_2_ illustrated reduced FCG rates that were marginally elevated above those in a pure nitrogen atmosphere. In higher stress domains (ΔK > 30 MPa·m^1/2^), the FCG rates swiftly surged and aligned with those in pure hydrogen environments. This indicated that at lower stress intervals (20~30 MPa·m^1/2^), 2.5% CO_2_ + 35 ppm O_2_ could enhance the fatigue performance of L360 pipeline steel. This may have been due to the suppressive collaboration of both impurity gases on hydrogen’s detrimental influence on fatigue damage. It became evident that the FCG rate was tantamount to that under a hydrogen environment in domains of heightened stress, suggesting that hydrogen’s influence at these elevated stress levels became the predominant factor directing the fatigue crack growth behavior of L360 steel.

### 3.2. Microscopic Fatigue Morphology

**Figure 6:**
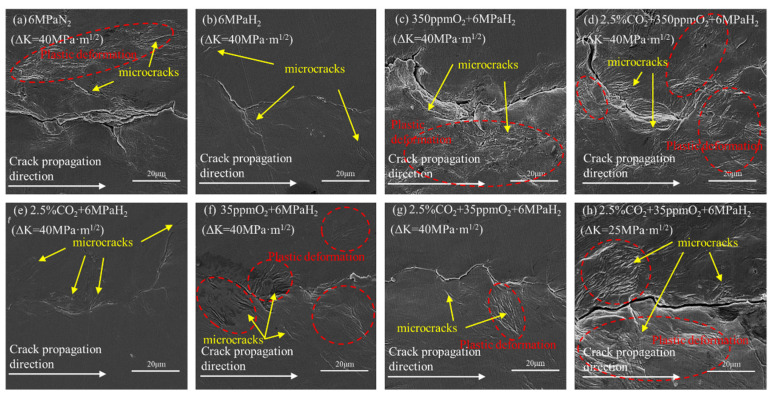
Microscopic view of the area around the crack tip of CT samples of L360 pipeline steel in seven environments.

**Figure 7:**
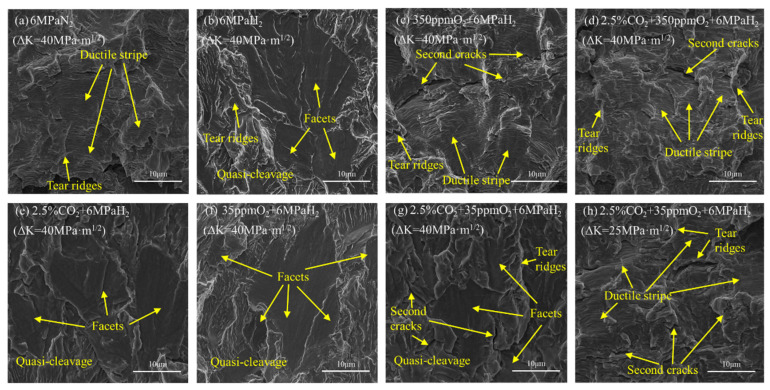
Fracture morphology of CT specimens of L360 pipeline steel in fatigue crack propagation experiments under various impurity environments (crack direction from bottom to top).

Figure 6 depicts the microscopic topography surrounding the crack-tip area of the L360 pipeline steel CT specimens in mixed gas environments. We discovered that the crack trails in a nitrogen atmosphere exhibited pronounced plastic deformation (Figure 7a); numerous distinct striated slip traces were apparent near the crack, yet these traces were confined to the vicinity of the fracture [28,29]. This suggested that during the fatigue crack growth (FCG) process, only the immediate peripheral region of the crack (where the stress was relatively concentrated) was involved in crack propagation. Figure 6c,d reveal that the crack and adjacent slip traces in the 350 ppm O_2_ + 6 MPa H_2_ group and the 2.5% CO_2_ + 350 ppm O_2_ + 6 MPa H_2_ group highly resembled the deformation observed within the nitrogen group, with evident plastic shaping. This indicated that the surface cracks predominantly propagated through ductile fracture mechanisms. Figure 6b,e,f demonstrate that there were exceedingly few slip bands around the crack path and minimal plastic zones in the environments of 6 MPa H_2_ + 35 ppm O_2_, 6 MPa H_2_, and 2.5% CO_2_ + 6 MPa H_2_. This signified that the crack had propagated in a brittle manner, devoid of extensive plastic deformation characteristics [30,31,32]. Figure 6g,h illustrate the fracture paths observed in the 2.5% CO_2_ + 35 ppm O_2_ + 6 MPa H_2_ group at stress intensity factor ranges of ΔK = 40 MPa·m^1/2^ and ΔK = 25 MPa·m^1/2^, respectively. There were fewer slip traces and less plastic shaping at higher values of ΔK, whereas significant plastic deformation was present at lower values of ΔK. This suggested the occurrence of ductile fractures under these conditions.

Figure 6 displays the fatigue fracture morphology of the material under a mixed gas atmosphere upon parting the specimen along the crack line. In a nitrogen environment, the fracture of the material was ductile and manifold tearing ridges and microcracks were visible [33] (Figure 7a). In contrast, the fracture surface within a hydrogen environment exhibited quasi-cleavage characterized by distinctive river-pattern markings and quasi-cleavage planes, a typical brittle fracture morphology [34]. The fractures in environments of 2.5% CO_2_ + 6 MPa H_2_ and 2.5% CO_2_ + 35 ppm O_2_ + 6 MPa H_2_ also exhibited quasi-cleavage planes indicative of brittle fractures, suggesting that neither 2.5% CO_2_ nor 35 ppm O_2_ inhibited the material’s susceptibility to brittle cracking in hydrogen environments. Conversely, in environments of 350 ppm O_2_ + 6 MPa H_2_ and 2.5% CO_2_ + 350 ppm O_2_ + 6 MPa H_2_, the fracture morphology of the material resembled the ductile fractures observed in nitrogen, with pronounced ductile striations and numerous microcracks distributed across the fracture surface [35]. This highlighted the strong mitigating effect of 350 ppm O_2_ on hydrogen embrittlement and its ability to overshadow the promoting effect of 2.5% CO_2_ on the embrittlement of the material. The fatigue crack growth rate of the material in the environment of 2.5% CO_2_ + 35 ppm O_2_ + 6 MPa H_2_ demonstrated a discernibly accelerated behavior within a selected range of ΔK. The fracture of the material was a brittle quasi-cleavage at ΔK = 40 MPa·m^1/2^, whereas ductile striations were present in the fracture at ΔK = 25 MPa·m^1/2^. This indicated that the material exhibited hydrogen embrittlement effects only at high ΔK levels, with the synergistic influence of 2.5% CO_2_ and 35 ppm O_2_ becoming more apparent as ΔK increased.

### 3.3. Results of Hydrogen Gas Permeation

Our evaluation of the material’s hydrogen permeation characteristics hinged on two pivotal indices: the breakthrough time for hydrogen to traverse the material and the steady-state current during the phase of stable diffusion. The breakthrough time was the interval from the initial contact of hydrogen gas on one side to its emergence on the opposite side of the material. This phase, represented on the hydrogen permeation curve from the commencement of hydrogen introduction to the precursor of the current’s ascent, chiefly reflected the rate at which hydrogen was adsorbed and dissociated on the surface at the beginning of permeation, the magnitude of the energy barrier to break through the surface to enter the material’s interior, and the resistance to diffusion within the sample. The steady-state current was the maximal current value corresponding with the phase of stable diffusion during the hydrogen permeation process and was represented by a stable current value after the maximum on the permeation curve. This parameter primarily elucidated the diffusion rate of hydrogen in a steady state, the driving concentration values of surface hydrogen, and the coverage rate of hydrogen at the surface.

**Figure 8:**
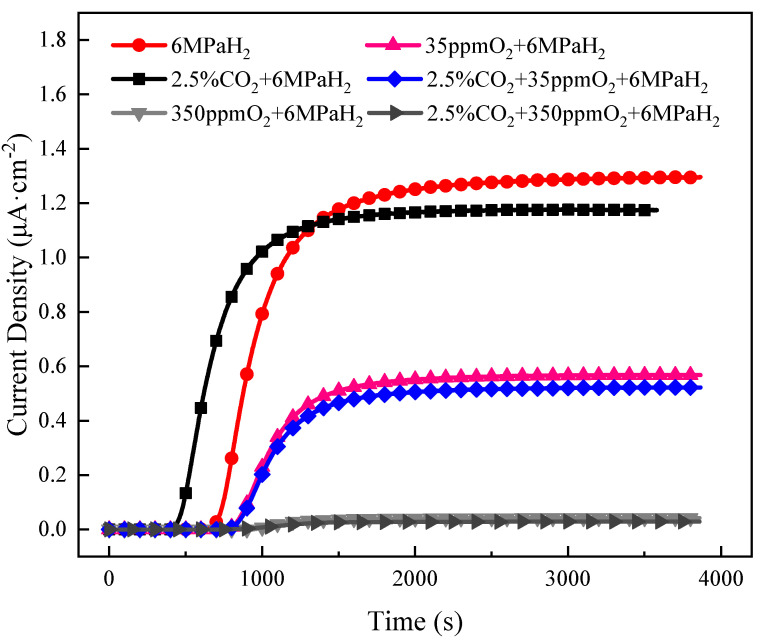
Gas hydrogen permeation curve.

Figure 8 reveals the hydrogen permeation results for L360 in mixed gas environments. When evaluated in conjunction with the breakthrough times and steady-state current values listed in Table 3, it became apparent that the inclusion of CO_2_ in the hydrogen gas abbreviated the material’s breakthrough time by 150 s. This indicated that CO_2_ could diminish the diffusion activation energy required for hydrogen to infiltrate the material’s interior. The slight reduction in the steady-state current suggested that CO_2_’s impact on the adsorption and dissociation of surface hydrogen was minimal, but it continued to occupy certain surface spots of the material, thus decreasing the active adsorption sites for hydrogen. The material’s breakthrough time extended with the addition of O_2_ to the hydrogen gas. The breakthrough time increased from 571 s in a pure H_2_ environment to 834 s with the inclusion of 35 ppm O_2_, and the steady-state current decreased to half its original value. This indicated that O_2_ had a substantial inhibitory effect on the adsorption of hydrogen on the material’s surface [36]. The hydrogen permeation curve almost lost its signal when the O_2_ concentration was increased to 350 ppm; this extended the breakthrough time to 963 s and reduced the steady-state current to one-thirtieth of its original figure. This suggested that almost all the hydrogen adsorption sites on the surface were occupied by O_2_, leaving fewer sites available for hydrogen adsorption. When CO_2_ and O_2_ were both introduced into the hydrogen gas, we observed that O_2_’s role was predominant. Thus, the effect of CO_2_ on reducing the diffusion barrier for hydrogen was almost insignificant.

## 4. Discussion

### 4.1. Hydrogen Acceleration of Fatigue Crack Propagation

In fatigue crack growth experiments with compact tension (CT) specimens in pure nitrogen, cyclic strain loading induces a stress concentration at the crack tip. This forms slip bands that relax the stress at the crack tip and enhance the resistance to crack propagation. Dislocations accumulate, leading to crack-tip blunting and the eventual cessation of crack growth [37]. The crack may propagate once more during subsequent stress cycles.

Conversely, in CT specimens under pure hydrogen, hydrogen plays a dominant role in the evolution of material microstructures. The hydrogen-enhanced localized plasticity (HELP) mechanism [38,39,40,41,42] suggests that hydrogen around dislocations shields the elastic stress field produced by dislocations, diminishing the interaction between dislocations and obstacles. This enables dislocation movement at lower stresses, promoting dislocation migration [33]. Consequently, hydrogen atoms in the grains congregate at high-stress concentration areas such as crack tips and grain boundaries because of rapid and constant dislocation movements during plastic deformation. Under cyclic stress loading, numerous slip bands form at the crack tip. This results in a significant accumulation of hydrogen atoms when exposed to a hydrogen environment as newly formed dislocations at the surface trap many hydrogen atoms. The occurrence of both phenomena leads to a significant concentration of hydrogen at the crack tip. The hydrogen-enhanced decohesion (HEDE) mechanism [38,39,43] posits that the presence of hydrogen atoms reduces the bonding strength between adjacent atoms, visibly increasing the propensity for crack initiation in these high-stress concentration areas under external forces. As the crack propagates along slip bands to the next grain boundary, hydrogen segregation occurs. This promotes crack propagation to an extent. A smooth cleavage morphology is observed under a hydrogen environment, indicating a transition from ductile fractures to brittle fractures in L360 steel under a nitrogen environment.

### 4.2. Effects of CO_2_ and O_2_ on the Hydrogen Embrittlement Behavior of L360 Pipeline Steel

When the gas composition was 2.5% CO_2_ + 6 MPa H_2_, the fatigue crack propagation rate at this time was comparable with that of H_2_ (as indicated by the curve in Figure 5). At a lower ΔK, its fatigue crack propagation rate was slightly higher than H_2_. Figure 7e reveals that there were slightly more decohesion fracture planes than for H_2_, suggesting that CO_2_ only slightly promoted the hydrogen embrittlement behavior of L360 pipeline steel in a high-pressure H_2_ environment. CO_2_ can strengthen the adsorption of H_2_ and accelerate the dissolution (migration from surface to subsurface) of the H atom when CO_2_ is adsorbed on the iron surface. Due to the fast adsorption of H_2_ itself, the promoted H dissolution rate by CO_2_ is the major reason for the enhanced hydrogen uptake and HE of the steel [21]. As illustrated in Table 3, the permeation of hydrogen through gas is significantly reduced by the presence of CO_2_. This indicates that during the growth of a crack, the presence of CO_2_ increases the concentration of hydrogen at the crack tip, resulting in a higher rate of material crack expansion.

We observed that the fatigue crack propagation rate in the environment of 350 ppm O_2_ + 6 MPa H_2_ was much lower than that in the 35 ppm O_2_ + 6 MPa H_2_ environment (Figure 5) and was similar to the fatigue crack propagation rate in a nitrogen environment. This suggested that ductile fractures might primarily occur at this time. The fatigue crack propagation rate in the 35 ppm O_2_ + 6 MPa H_2_ environment was similar to that in the hydrogen environment, suggesting that brittle fractures might primarily occur. Comparing the transverse fracture paths of the two experimental groups (Figure 6c,f), we observed that there were many slip traces around the crack in the environment of 350 ppm O_2_ + 6 MPa H_2_, with a significant plastic zone indicating a ductile fracture. In contrast, there were far fewer slip traces around the crack in the 35 ppm O_2_ + 6 MPa H_2_ environment than in the 350 ppm O_2_ + 6 MPa H_2_ environment, indicating a brittle fracture. When comparing the microscopic fracture morphologies (Figure 7c,f), a large number of fatigue striations and river-pattern ductile fracture traces were observed in the 350 ppm O_2_ + 6 MPa H_2_ environment. These were similar to those in the nitrogen environment, indicating that the fracture was primarily ductile. A large number of cleavage platforms were observed in the 35 ppm O_2_ + 6 MPa H_2_ environment fracture morphology, which was different from the nitrogen environment. The appearance of cleavage platforms was related to hydrogen because hydrogen restricted the dislocation slip during deformation, reducing the material’s plastic deformation capability and thus evolving the material’s fracture mode into one that was primarily brittle.

We observed that 350 ppm O_2_ inhibited the effect of hydrogen on accelerating fatigue crack propagation in L360 pipeline steel, whereas 35 ppm O_2_ did not have this inhibitory effect. Considering the hydrogen permeation results presented in Figure 8 and Table 3, we inferred that the reason for the above phenomena was that when the L360 pipeline steel underwent fatigue crack propagation in the environment of 350 ppm O_2_ + 6 MPa H_2_, both O_2_ and H_2_ diffused to the crack-tip surface and adsorbed there. At this time, O_2_ occupied the adsorption sites of H_2_ because of the high concentration of O_2_ and its stronger adsorption capability than H_2_, driving it away from the crack tip. This resulted in the interaction between H and the dislocation at the crack tip being avoided, ultimately reducing the hydrogen embrittlement sensitivity of the L360 steel [44]. When the L360 pipeline steel underwent fatigue crack propagation in the environment of 35 ppm O_2_ + 6 MPa H_2_, O_2_ and H_2_ also diffused to the crack tip surface and adsorbed there. At this time, the oxygen content was too low to completely occupy the adsorption sites of H_2_, allowing H to enter the interior of the specimen. This disrupted the interatomic forces and accelerated the fatigue crack propagation rate. When a certain threshold was reached, the crack propagation rate was so fast that the oxygen could not occupy the adsorption sites of H_2_ at the crack tip. The crack continued to propagate and generated a new crack tip, thereby negating the inhibitory effect of oxygen on the hydrogen embrittlement behavior of the pipeline steel.

### 4.3. Combined Effects of CO_2_ and O_2_ on the Hydrogen Embrittlement Behavior of L360 Pipeline Steel

When the gas composition was 2.5% CO_2_ + 350 ppm O_2_ + 6 MPa H_2_, the fatigue crack growth rate was far lower than that under pure hydrogen and was similar to the fatigue crack growth rate under nitrogen (Figure 5). This indicated that L360 pipeline steel might not exhibit hydrogen embrittlement under these conditions. This perspective is reflected in Figure 6d and Figure 7d. A large number of plastically deformed zones could be observed around the crack when observing the transverse fracture path. Fatigue striations were discovered during the microscopic fracture morphology observations. These were similar to the fractures in a pure nitrogen environment. From this, we concluded that the fracture mode of L360 pipeline steel at this time was primarily ductile fractures.

From the results of hydrogen permeation, we concluded that the adsorption capacities of H_2_, CO_2_, and O_2_ increased in that order. At an oxygen content of 350 ppm, the strong adsorption capability of oxygen led to the newly formed surfaces at the crack tip being completely covered by O_2_ because of the sufficiently high oxygen content, even if the three gases were evenly distributed at the crack tip at the beginning. This indicated that all adsorption sites were occupied by O_2_. At this time, CO_2_ could not be captured by the large number of dislocations generated at the crack tip, preventing CO_2_ from promoting the capture and absorption of hydrogen at the crack tip. Hence, the fractures were primarily ductile during this process.

When the gas composition was 2.5% CO_2_ + 35 ppm O_2_ + 6 MPa H_2_, we observed that the fatigue crack growth rate under low-stress conditions was slightly higher than the FCG rate of the pure nitrogen group (Figure 5). Under high-stress conditions, the FCG rate was similar to that of the pure hydrogen group. Figure 6 and Figure 7 also reveal two distinct regions, indicating that the fracture modes in these regions were different.

In Figure 6g, local slip marks can be observed around the crack. These were not as numerous as those in the nitrogen environment, suggesting that hydrogen embrittlement may still have occurred. By pulling the CT specimen along the crack direction and observing the fracture morphology (Figure 7g,h), we noted that the fracture mode was mainly ductile fatigue fractures at a low ΔK, whereas the fracture mode was brittle at a high ΔK and displayed hydrogen embrittlement behavior with a clear difference between the two regions. The observed inhibitory effect of O_2_ + CO_2_ and the mechanism by which O_2_ inhibited the acceleration of the fatigue crack growth by hydrogen were explored, as expounded by Somerday et al. [17].

In the 2.5% CO_2_ + 35 ppm O_2_ + 6 MPa H_2_ gas group, at a low ΔK, and when the crack growth rate was low, the three gases were evenly distributed at the crack tip. O_2_ had sufficient time to occupy the adsorption sites of H_2_ adsorbed at the crack tip and replace the H_2_ because of the stronger adsorption capability of O_2_ and the slow crack growth rate. Spare adsorption sites were available because of the low oxygen content. CO_2_ gas was present at this time; as CO_2_ has a stronger adsorption capability than H_2_, it also replaced the H_2_ adsorbed at the crack tip and occupied the adsorption sites. This prevented the interaction between hydrogen and the dislocations at the crack tip, reducing the impact of hydrogen on the fatigue crack growth of L360 steel at a low ΔK.

As ΔK increased and the fatigue crack growth rate accelerated to reach a certain threshold, CO_2_ and O_2_ did not have sufficient time to occupy all H_2_ adsorption sites [45,46]. In each loading cycle, the newly formed crack tip surfaces were not completely covered by impurity gases. This allowed the hydrogen atoms to enter from the crack tip surface and accelerate the fatigue crack growth. At a high ΔK, the three gases were evenly adsorbed at the crack tip. However, O_2_ and CO_2_ did not have sufficient time to replace the H_2_ adsorbed on the old crack tip because of the larger fatigue crack growth rate. This allowed H to enter the crack tip surface and be fully adsorbed. This reduced the binding force between the atoms at the crack tip, causing the crack to accelerate its expansion because of H’s action and form a new crack tip. This indicated that O_2_ and CO_2_ had no impact on the hydrogen embrittlement behavior of L360 pipeline steel at a high ΔK.

## 5. Conclusions

In this study, we examined the effects of impurity gases on the hydrogen embrittlement behavior of L360 pipeline steel under various conditions (6 MPa N_2_, 6 MPa H_2_, 350 ppm O_2_ + 6 MPa H_2_, 35 ppm O_2_ + 6 MPa H_2_, 2.5% CO_2_ + 350 ppm O_2_ + 6 MPa H_2_, 2.5% CO_2_ + 35 ppm O_2_ + 6 MPa H_2_, and 2.5% CO_2_ + 6 MPa H_2_) using fatigue crack growth experiments. We explored the mechanism of the collective influence of various impurity gases on L360 pipeline steel in a high-pressure H_2_ environment. The principal conclusions were as follows.

(1) The L360 pipeline steel exhibited a significant hydrogen embrittlement phenomenon in a high-pressure hydrogen environment. The addition of 350 ppm O_2_ in a 6 MPa H_2_ environment decreased the fatigue crack growth rate to the level observed in the N_2_ environment. When the O_2_ concentration was reduced to 35 ppm, the fatigue crack growth rate was similar to that in a pure hydrogen environment. This indicated that O_2_ could suppress the hydrogen embrittlement sensitivity of pipeline steel in a high-pressure H_2_ environment and that this suppression effect was concentration-dependent.
(2) CO_2_ had a slight promoting effect on the hydrogen embrittlement behavior of L360 pipeline steel in a high-pressure H_2_ environment. CO_2_ enhanced the material’s hydrogen permeation by shortening the penetration time of hydrogen through the material. Conversely, O_2_ inhibited the material’s hydrogen permeation, not only by extending the breakthrough time but also by reducing the material’s steady-state current value. As the O_2_ concentration increased, its inhibitory effect strengthened; a 30-fold signal reduction was observed at 350 ppm. When O_2_ and CO_2_ coexisted in hydrogen gas, O_2_ masked the promoting effect of CO_2_ on hydrogen permeation. This demonstrated that O_2’_s adsorption capacity surpassed that of CO_2_ on the surface of L360 steel.
(3) In a 6 MPa H_2_ environment, when 35 ppm O_2_ and 2.5% CO_2_ were simultaneously added, the fatigue crack growth rate of L360 pipeline steel was reduced in low-stress conditions, producing lower fatigue crack growth rates. The fracture mode was predominantly ductile. Under high-stress conditions where the fatigue crack growth rate was higher, the inhibitory effect of 35 ppm O_2_ and 2.5% CO_2_ almost disappeared, and the fracture mode became brittle. This was attributed to the fact that at lower crack growth rates, 35 ppm O_2_ and 2.5% CO_2_ had ample time to replace the H_2_ molecules on the metal surface, thereby reducing the hydrogen coverage on the metal surface. At higher crack growth rates, the gas mixture did not have sufficient time to replace the H_2_ molecules on the metal surface.

## Figures and Tables

**Table 1:** Chemical composition of L360 steel (mass %).

Material	C	Si	Mn	P	S	Cr	Cu	Mo	V	Fe
L360	0.10	0.33	1.33	0.01	0.002	0.10	0.038	0.10	0.05	Bal.

**Table 2:** Gas mixture composition.

1	2	3	4	5	6	7
6 MPa H_2_	6 MPa N_2_	2.5% CO_2_ + 6 MPa H_2_	350 ppm O_2_ + 6 MPa H_2_	35 ppm O_2_ + 6 MPa H_2_	2.5% CO_2_ + 350 ppm O_2_ + 6 MPa H_2_	2.5% CO_2_ + 35 ppm O_2_ + 6 MPa H_2_

**Table 3:** Results relating to the hydrogen permeation of gases.

Environment	Penetration Time $t_{b}$ (s)	Steady-State Current $I_{\infty }$ $\left(\mu A/cm^{2}\right)$
6 MPa H_2_	571 ± 4	1.2637 ± 0.0103
2.5% CO_2_ + 6 MPa H_2_	421 ± 5	1.1731 ± 0.0311
350 ppm O_2_ + 6 MPa H_2_	963 ± 7	0.0411 ± 0.0004
35 ppm O_2_ + 6 MPa H_2_	834 ± 9	0.5674 ± 0.0225
2.5% CO_2_ + 350 ppm O_2_ + 6 MPa H_2_	819 ± 7	0.5217 ± 0.0009
2.5% CO_2_ + 35 ppm O_2_ + 6 MPa H_2_	950 ± 5	0.0289 ± 0.0006

## Data Availability

The data presented in this study are available upon request from the corresponding author.
